# Hydrogen and Ammonia Co-Adsorption on *M*(1 1 1) and Pd_3_*M*(1 1 1) (*M* = Pd, Ru, Ag, Au, Cu) Surfaces

**DOI:** 10.3390/membranes15050135

**Published:** 2025-05-01

**Authors:** Didrik R. Småbråten, Marie D. Strømsheim, Thijs A. Peters

**Affiliations:** 1 Department of Sustainable Energy Technology, SINTEF Industry, 0314 Oslo, Norway; thijs.peters@sintef.no; 2 Hydrogen Mem-Tech AS, 7038 Trondheim, Norway; marie.stromsheim@hydrogen-mem-tech.com

**Keywords:** hydrogen, ammonia, modelling, co-adsorption

## Abstract

Ammonia (NH_3_) represents a promising zero-emission fuel in hydrogen fuel cells. Membrane reactors for NH_3_ decomposition based on Pd-alloys have demonstrated high NH_3_ conversion, high hydrogen diffusivity, and high hydrogen selectivity, which allows for the production of high-purity H_2_ without the need for gas separation or purification. However, it is observed that Pd-alloy membranes are to a various degree prone to H_2_ flux inhibition in the presence of NH_3_. Hence, finding proper means to tailor the surface adsorption properties through, e.g., alloying is imperative to further improve the technology. In the current work, hydrogen and ammonia co-adsorption phenomena on *M*(1 1 1) and Pd_3_*M*(1 1 1) (*M* = Pd, Ru, Ag, Au, Cu) surfaces are studied using density functional theory calculations. It is shown that the surface adsorption properties are strongly dependent on the surface composition, which can be linked to the corresponding electronic structure at the membrane surface.

## 1. Introduction

Due to its high volumetric energy density, low cost and ease of liquefaction, storage, and transportation [1], ammonia (NH_3_) represents a highly promising zero-emission fuel in the maritime and heavy transport sector. NH_3_ can be converted directly to electricity in, e.g., combustion engines [2,3,4,5,6,7,8,9] and gas turbines [10], or used as fuel in fuel cells like solid oxide fuel cells (SOFCs), alkaline fuel cells (AFCs), or indirectly, i.e., after decomposition and purification, in polymer electrolyte membrane fuel cells (PEMFCs) [11,12]. Fuel cell technologies show several advantages over combustion engines and gas turbines, such as higher efficiency that is independent of plant scale, and that they are modular [6]. SOFCs show the overall highest efficiency, can operate in external or internal cracking modes, and do not require comprehensive fuel purification [6,13].

Pd-based membrane-enhanced reactors have been considered a promising system to efficiently recover the H_2_ stored in NH_3_ that would inherently produce high-purity H_2_ through the membranes, avoiding the need for an additional hydrogen separation unit [14,15,16,17,18,19,20]. Furthermore, full ammonia conversion could be obtained, minimising the need for the downstream cleaning of unconverted ammonia [15,17,18,21,22,23]. However, reports show that NH_3_ present in the gas feed limits membrane performance, even for concentrations of NH_3_ in the gas feed as low as 10 to 500 ppm [19,20]. To elucidate this, Peters et al. [20] carried out density functional theory (DFT) calculations to study the competitive adsorption of H and NH_3_ on the surface of the employed Pd-Ag membranes. Their computational results concluded that H_2_ flux inhibition in the presence of NH_3_ could not be explained by a lowering of the hydrogen surface coverage due to the competitive adsorption of NH_3_-related species. Building on the study by Peters et al. [20], and previous knowledge on the competitive adsorption of H and NH_3_ [20], the competitive adsorption of H and S [24], and the co-adsorption of H and CO [25,26] on Pd(1 1 1) and Pd_3_Ag(1 1 1), we further elaborated on the co-adsorption of H and NH_3_ on Pd(1 1 1) and Pd_3_Ag(1 1 1) surfaces in our recent work [27]. Here, we found that both hydrogen surface coverage and dissociation energetics were inhibited in the presence of NH_3_ on the surface, and the possible segregation of Ag towards the surface in the presence of NH_3_ was suggested, which could explain the observed hydrogen flux inhibition in the presence of NH_3_.

Our previous work [27] established a methodology to study adsorption thermodynamics, adsorbate–adsorbate interactions during co-adsorption, adsorption-induced surface segregation effects, and hydrogen dissociation energetics for hydrogen and ammonia-related species using DFT. In the following work, the methodology is applied to other experimentally relevant *M*(1 1 1) and Pd_3_*M*(1 1 1) (*M* = Pd, Ru, Ag, Au, Cu) surfaces.

## 2. Methods

### 2.1. DFT Calculations

DFT calculations were made using the projector augmented wave (PAW) method as implemented in VASP [28,29,30]. Pd (4p^6^, 4d^9^, 5s^1^), Ru (4p^6^, 4d^7^, 5s^1^), Ag (4p^6^, 4d^10^, 5s^1^), Au (5d^10^, 6s^1^), Cu (3p^6^, 3d^10^, 4s^1^), H (1s^1^), and N (2s^2^, 2p^3^) were treated as valence electrons, using the GGA-PBE functional [31]. The plane-wave cutoff energy was set to 500 eV, with an electronic convergence of 10^−6^ eV. Geometry optimization was performed until the residual forces on all atoms were within 0.02 eV Å^−1^, with the two bottom layers fixed to bulk positions. When calculating the vibrational frequencies the adsorbates were further relaxed with a force criterion of 10^−3^ eV Å^−1^. Vibrational properties were determined by the finite displacement method using four displacements (±0.015 Å and ±0.30 Å) along each of the Cartesian directions. The minimum energy paths (MEP) for hydrogen dissociation were determined by the climbing image nudged elastic band (cNEB) method [32,33]. 11 intermediate images were used, with a force criterion of 0.05 eV Å^−1^.

The (1 1 1) surfaces were modelled by slabs with a thickness of 7 atomic layers and a 25 Å vacuum layer. Dipole corrections were included. The surface coverages, θ, were defined as the number of adsorbed species per surface metal atom, i.e., one adsorbed species per surface metal atom corresponds to full surface coverage of θ = 1. Adsorption was considered in *p*(2 × 2) supercells containing 4 metal atoms per layer. A Γ-centred 6 × 6 × 1 *k*-point grid was used for the *p*(2 × 2) slabs. Adsorption induced surface segregation effects were determined from the relative energies for adsorption on model structures with varying number of Pd and *M* atoms within the four topmost layers. In the following, the seven structures described in Refs. [26,27] were investigated. The alloys were modelled assuming a nominal stoichiometry of Pd_3_*M*.

### 2.2. Thermodynamic Modelling

The adsorption thermodynamics at finite temperatures *T* were determined by the Gibbs adsorption energies of the equilibria in Equations (6)–(10) according to

(1) $$\Delta G_{ads}=\Delta H_{ads}-T\Delta S_{ads}$$

Here, $\Delta H_{ads}$ is the adsorption enthalpy and $\Delta S_{ads}$ the adsorption entropy. The adsorption enthalpies were calculated by

(2) $$\Delta H_{ads}=\Delta E_{ads}^{DFT}-\left(E_{gas}^{DFT}+H_{gas}^{ref}\right)+\Delta ZPE$$

where $\Delta E_{ads}^{DFT}$ is the DFT-calculated total energy differences for the slabs with and without adsorbed species, $E_{gas}^{DFT}$ is the DFT-calculated total energy of gaseous H_2_ or NH_3_, and $H_{gas}^{ref}$ denotes the reference states for gaseous species at finite temperatures taken from thermochemical data [34]. Zero-point energy (ZPE) contributions were calculated by the vibrational frequencies of the adsorbed species, $\nu_{i}$, according to

(3) $$ZPE=\sum_{i}\frac{h\nu_{i}}{2}$$

The adsorption entropy of NH_3_ was obtained from the empirical relations by Campbell and Sellers [35]. For the other species, the adsorption entropies were calculated as

(4) $$\Delta S_{ads}\left(T\right)=S_{ads}^{vib}\left(T\right)-S_{gas}^{ref}(T)$$

where $S_{ads}^{vib}$ is the vibrational entropy of the adsorbed species calculated from their vibrational frequencies $\nu_{i}$ according to

(5) $$S_{ads}^{vib}=k\sum_{i}\left(\frac{\beta_{i}}{\exp\left(\beta_{i}\right)-1}-\ln\left(1-\exp\left(-\beta_{i}\right)\right)\right),\beta_{i}=h\nu_{i}/k_{B}T,$$

and $S_{gas}^{ref}$ is the entropy of the gas molecules taken from thermochemical data [34].

The adsorption thermodynamics for the hydrogen and NH_3_-related species were evaluated by the following equilibria [20,27]

(6) $$\frac{1}{2}H_{2}\left(g\right)+*⇌*H$$

(7) $$NH_{3}\left(g\right)+*⇌*N+\frac{3}{2}H_{2}\left(g\right)$$

(8) $$NH_{3}\left(g\right)+*⇌*NH+H_{2}(g)$$

(9) $$NH_{3}\left(g\right)+*⇌*NH_{2}+\frac{1}{2}H_{2}(g)$$

(10) $$NH_{3}\left(g\right)+*\;⇌\;*NH_{3}$$

where ∗ denotes a surface site or an adsorbed species on the surface. The equilibrium surface coverages $\theta_{i}$ were calculated by simultaneously solving the corresponding equilibrium constants

(11) $$K_{1}=\exp\left(-\frac{\Delta G_{1}^{ads}}{kT}\right)=\theta_{H}\theta_{v}^{-1}p_{H_{2}}^{-1/2}$$

(12) $$K_{2}=\exp\left(-\frac{\Delta G_{2}^{ads}}{kT}\right)=\theta_{N}\theta_{v}^{-1}p_{H_{2}}^{3/2}p_{NH_{3}}^{-1}$$

(13) $$K_{3}=\exp\left(-\frac{\Delta G_{3}^{ads}}{kT}\right)=\theta_{NH}\theta_{v}^{-1}p_{H_{2}}p_{NH_{3}}^{-1}$$

(14) $$K_{4}=\exp\left(-\frac{\Delta G_{4}^{ads}}{kT}\right)=\theta_{NH_{2}}\theta_{v}^{-1}p_{H_{2}}^{1/2}p_{NH_{3}}^{-1}$$

(15) $$K_{5}=\exp\left(-\frac{\Delta G_{5}^{ads}}{kT}\right)=\theta_{N}\theta_{v}^{-1}p_{NH_{3}}^{-1}$$

assuming that the total surface coverage is conserved according to $\sum_{i}\theta_{i}$ = 1. Here, $\theta_{v}$ refers to an empty surface site. Representative calculated adsorption atomic configurations for the different species are illustrated for Pd-terminated Pd_3_Au in Figure 1.

## 3. Results and Discussion

### 3.1. M(1 1 1) Surfaces

#### 3.1.1. Adsorption Thermodynamics

To investigate how different alloying could affect the adsorption properties, their respective pure metals were initially investigated as a reference point. The calculated adsorption energies $\Delta H^{ads}$, entropies $T\Delta S^{ads}$, energies $\Delta G^{ads}$, and vibrational frequencies $\nu_{i}$ for the different adsorbed species at 623 K at standard conditions ($p_{H_{2}}$ = $p_{NH_{3}}$ = $p^{0}$ = 1 bar) on the (1 1 1) surfaces for Pd, Ru, Ag, Au, and Cu are summarised in Table 1. H and NH_3_ show negative adsorption energies on Pd of −0.25 eV and −0.20 eV, respectively, while N, NH, and NH_2_ all show positive adsorption energies of 1.28 eV, 0.84 eV, and 0.70 eV, in line with previous work [27]. This indicates that H and NH_3_ will adsorb on the surface, while N, NH, and NH_2_ are repelled. Note that in Ref. [27], we evaluated the adsorption thermodynamics for a lower surface coverage of $\theta_{i}$ = 1/16. The slightly weaker NH_3_ adsorption reported here is due to the associated larger adsorbate–adsorbate interaction. Ru shows comparable adsorption energies for H and NH_3_ as Pd. However, N, NH, and NH_2_ all show less positive adsorption energies on Ru compared to on Pd. Furthermore, NH and NH_2_ show exothermic adsorption. This is in line with previous work, showing that NH_3_ is expected to dissociate on the Ru(0001) surface [36,37].

Ag and Au, on the other hand, show comparable adsorption thermodynamics, with positive adsorption energies for all species. Cu lies in between the extrema of Pd/Ru and Ag/Au, though it still has positive adsorption energies for all species. The adsorption energies for the pure metals are in line with previous work [27,38,39,40]. Note that in Refs. [38,39,40], free atoms and molecules of the adsorbates H, N, NH, NH_2_, and NH_3_ have been used as reference energies, compared to the equilibrium between H_2_ (g) and NH_3_ (g) described in Equations (6)–(10) used in the present work. These results suggest that Pd- and Ru-rich surfaces are expected to show comparable adsorption properties, Cu-rich surfaces intermediate adsorption properties, while Ag and Au on the surface could be detrimental for adsorption performance.

A comparison of the calculated adsorption thermodynamics for H and NH_3_ on Pd(1 1 1) using different DFT functionals including PBEsol [41], RPBE [42], revPBE [43], r^2^SCAN [44], DFT-D3(BJ) [45], and rev-vdW-DF2 [46,47,48] is shown in Table S3 in the Supplementary Information.

**Figure 2:**
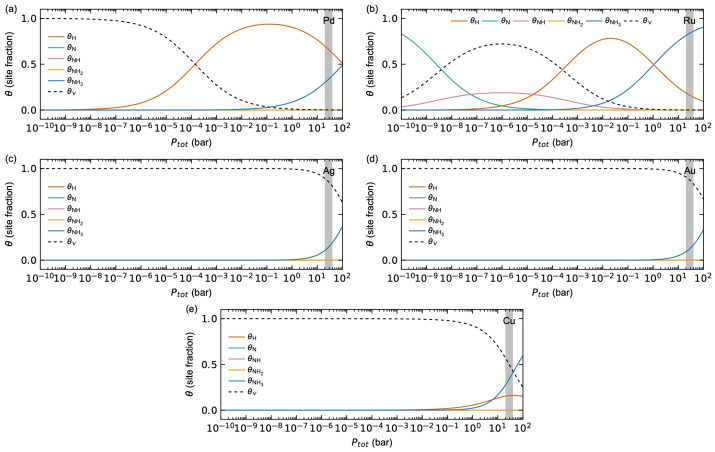
Calculated surface coverages *θ_i_* on the (1 1 1) surfaces of (**a**) Pd, (**b**) Ru, (**c**) Ag, (**d**) Au, and (**e**) Cu, assuming a gas composition of $x_{{\mathrm{NH}}_{3}}$ = $x_{N_{2}}$ = 0.2 and $x_{H_{2}}$ = 0.6 at *T* = 623 K. Typical operating conditions for the Pd-alloy membranes of 20–40 bar are marked in grey.

Figure 2 shows the calculated surface coverages for H, N, NH, NH_2_, NH_3_, and empty surface sites (*v*) as a function of total pressure $P_{tot}$ at 623 K, evaluated from the thermodynamic properties in Table 1. Here, a gas mixture corresponding to 60% H_2_, 20% NH_3_, and 20% N_2_ (inert) is assumed, mimicking a representative NH_3_ decomposition equilibrium. Focusing first on Pd, H starts to adsorb for $P_{tot}$ > 10^−7^, reaching a peak close to full saturation ($\theta_{H}$ ≈ 1) at $P_{tot}$ ≈ 10^−1^. Above this point, the competitive adsorption of H and NH_3_ is observed, indicated by an increase in $\theta_{NH_{3}}$ accompanied by a decrease in $\theta_{H}$. Ru shows a more complex evolution of surface coverage with respect to $P_{tot}$. For very low $P_{tot}$ < 10^−8^, the surface is governed by N. By increasing $P_{tot}$, $\theta_{NH}$ increases at the expense of $\theta_{N}$ reaching a maximum at $P_{tot}$ ≈ 10^−6^. Next, the surface becomes governed by H until a maximum is reached at around $P_{tot}$ ≈ 10^−2^–10^−2^. Finally, the surface becomes dominated by NH_3_ from $P_{tot}$ > 1. Ag and Au show no surface coverage of any of the species, except for NH_3_, which stars to adsorb for $P_{tot}$ > 1. Cu behaves similarly as Ag and Au, though with some additional surface coverage of H in line with the less positive hydrogen adsorption energy.

**Figure 3:**
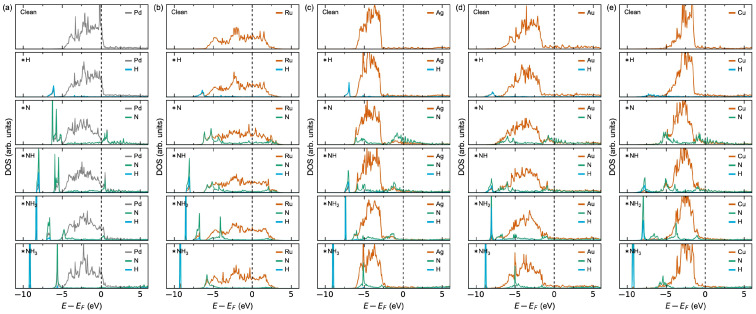
Atomically resolved electronic density of states (DOS) at the surface for a clean surface and with the adsorbates on (**a**) Pd, (**b**) Ru, (**c**) Ag, (**d**) Au, and (**e**) Cu. Only the DOS for the adsorbates and the nearest metal surface atoms are shown. The DOS are scaled per atom.

The thermodynamics can be reasoned from the calculated electronic density of states (DOS) upon adsorption plotted in Figure 3. For Pd, the large negative adsorption enthalpies of H and NH_3_ can be explained by the overlapping Pd and H or N states at the bottom of the valence band and the insignificant perturbation of the Pd states at the Fermi level [27]. Oppositely, the positive adsorption enthalpies for N and NH can be reasoned from a significant lowering of the Pd states at the Fermi level with an associated destabilisation effect [49]. The DOS for NH_2_ adsorption lies in between the two extrema, reflecting the corresponding intermediate adsorption energies. The tendency for strong adsorption on Ru can be explained already from the DOS for the clean surface, where the Fermi level lies within a high-density Ru *d*-band. Oppositely, the weak adsorption on Ag, Au, and Cu can be reasoned by the Fermi level being in a low-density *M* d-band in line with the *d*-band centre theory reported in the literature [50,51].

#### 3.1.2. Adsorbate–Adsorbate Interactions and Co-Adsorption Energetics

The adsorption thermodynamics described above show a significant expected equilibrium concentration for H and NH_3_ under the relevant operating conditions. Note, however, that these calculations do not take adsorbate–adsorbate interactions into consideration. Since adsorbate–adsorbate interactions can become significant for larger surface coverages, and in particular for larger adsorbates like NH_3_, we present in the following how the adsorption energies for H and NH_3_ are affected by significant adsorbate–adsorbate interactions explicitly.

**Figure 4:**
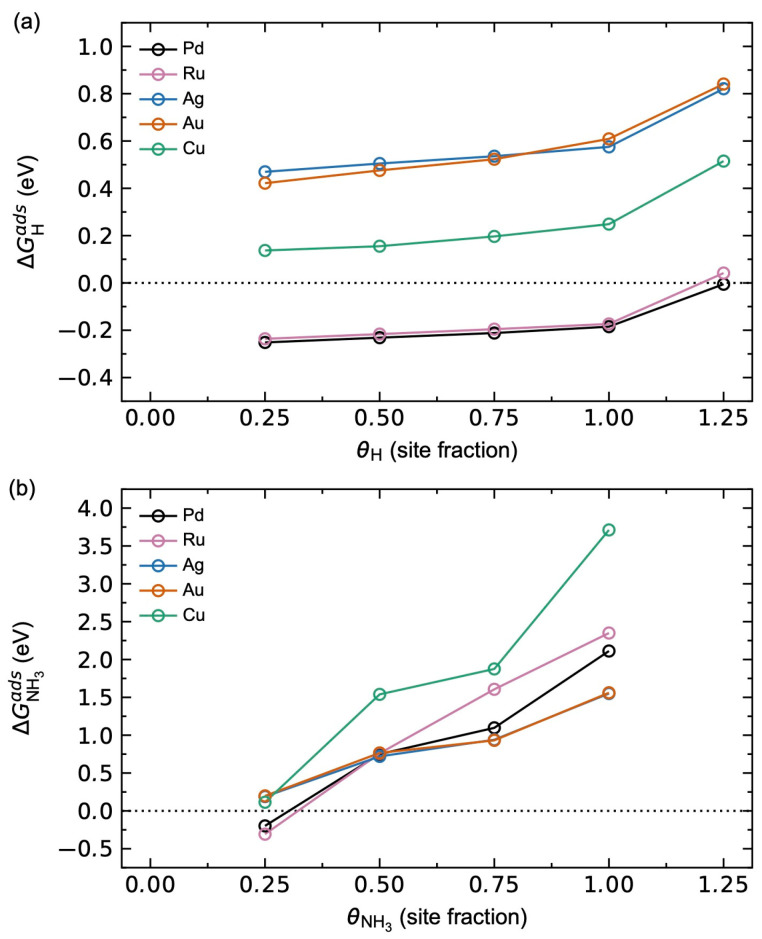
Calculated adsorption Gibbs energy for (**a**) H and (**b**) NH_3_ as a function of surface coverage *θ_i_* at T = 623 K and $p_{H_{2}}$ = $p_{{\mathrm{NH}}_{3}}$ = *p*^0^ = 1 bar on the pure metal surfaces.

The calculated adsorption energies for H and NH_3_ as a function of surface coverage are shown in Figure 4. The hydrogen adsorption in Figure 4a illustrates that the metals investigated can be divided into three groups. Pd and Ru both show negative $\Delta G_{H}^{ads}$ up to $\theta_{H}$ = 1, suggesting full hydrogen saturation in line with previous work [27]. Ag and Au show comparable and positive $\Delta G_{H}^{ads}$ for all $\theta_{H}$, while Cu lies in between the two extrema. The shapes of all the $\Delta G_{H}^{ads}$ profiles are, however, similar, where a sudden increase in $\Delta G_{H}^{ads}$ is observed for $\theta_{H}$ = 1.25. At this point, the hcp surface sites become occupied, where the destabilisation is due to the shorter H_fcc_-H_hcp_ distances (1.61 Å for Pd) compared to the longer H_fcc_-H_fcc_ distances (2.79 Å for Pd) for $\theta_{H}$ ≤ 1.

NH_3_ adsorption in Figure 4b shows a much steeper increase in adsorption energy with respect to surface coverage due to the significantly larger size of NH_3_ and corresponding larger adsorbate–adsorbate interactions, as previously reported [27]. Pd and Ru show an NH_3_ saturation coverage of 0.25, while Ag, Au, and Cu all show positive $\Delta G_{NH_{3}}^{ads}$, as described above. Ag and Au show comparable $\Delta G_{NH_{3}}^{ads}$, with near complete overlap between the values for the two alloying elements.

**Figure 5:**
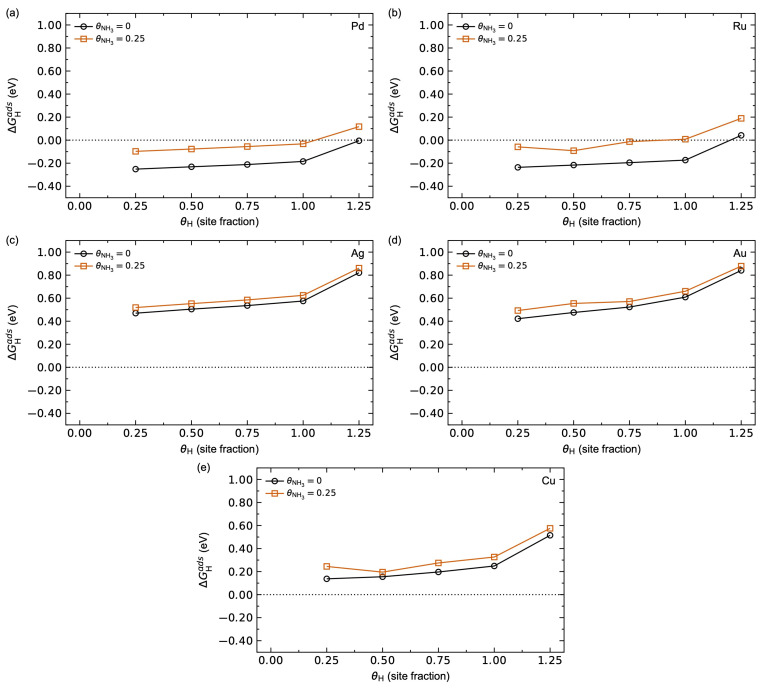
Calculated hydrogen adsorption energy for a clean surface (black) and with $\theta_{{\mathrm{NH}}_{3}}$ = 0.25 pre-adsorbed on the surface (red) for (**a**) Pd, (**b**) Ru, (**c**) Ag, (**d**) Au, and (**e**) Cu as a function of hydrogen surface coverage *θ*_H_ at *T* = 623 K and $p_{H_{2}}$ = $p_{{\mathrm{NH}}_{3}}$ = *p*^0^ = 1 bar.

Finally, the adsorption energies for hydrogen on a clean surface to a surface with $\theta_{NH_{3}}$ = 0.25 pre-adsorbed on the surface are compared in Figure 5. The destabilisation of hydrogen adsorption of ~0.2 eV for Pd and Ru is observed in Figure 5a and b, respectively. The destabilisation effect is less pronounced for Ag, Au, and Cu, which can be attributed to their corresponding weaker NH_3_ adsorption.

#### 3.1.3. Hydrogen Diffusion Energetics

**Figure 6:**
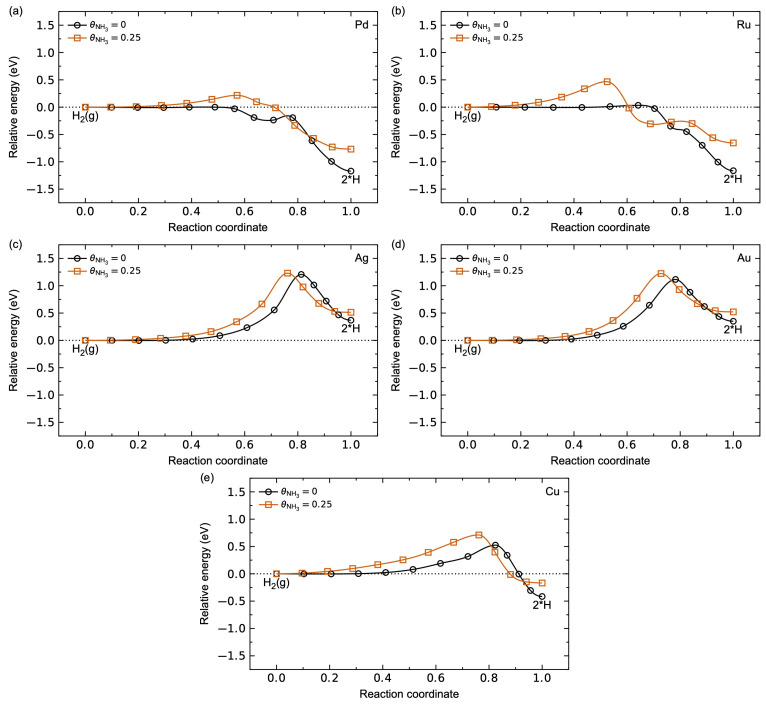
Calculated minimum energy path (MEP) for hydrogen dissociation for a clean surface (black) and with $\theta_{NH_{3}}$ = 0.25 pre-adsorbed on the surface (red) for (**a**) Pd, (**b**) Ru, (**c**) Ag, (**d**) Au, and (**e**) Cu.

The calculated minimum energy path (MEP) for hydrogen dissociation without and with NH_3_ pre-adsorbed on the metal surfaces is plotted in Figure 6. The H_2_ dissociation is a non-activated process on the Pd and Ru surfaces, apparent from the lack of an energy barrier relative to H_2_. As previously reported [27,52,53], Pd shows a local energy minimum along the MEP, corresponding to a metastable physiosorbed H_2_ molecule on a top site, with a small energy barrier (~0.04 eV) for the splitting of the H-H bond. Ru, on the other hand, does not show this local energy barrier. This indicates that the Ru(1 1 1) surface is more active towards H_2_ dissociation compared to Pd(1 1 1). Ag and Au, on the other hand, show significant energy barriers for the hydrogen dissociation of 1.21 eV and 1.12 eV, respectively. The corresponding dissociation mechanism goes through H_2_ physiosorbed on a hollow site rather than physiosorbed H_2_ on a top site as for Pd and Ru. Interestingly, while the adsorption enthalpy for H is negative on Cu, the dissociation has an energy barrier of 0.53 eV due to a dissociation path comparable to Ag and Au. This suggests that the emerging energy barriers for Ag, Au, and Cu are due to the hydrogen adsorption energetics on the surface, as described above.

With NH_3_ pre-adsorbed on the surface, the hydrogen dissociation becomes an activated process on all surfaces, with a positive energy barrier relative to H_2_ along all the MEP. Pd and Ru show energy barriers of 0.22 eV and 0.47 eV, respectively, while the energy barrier on Ag is insensitive to the presence of NH_3_. The emerging energy barrier on Pd in the presence of NH_3_ is described in our previous work [27]; the dissociation goes through a physiosorbed H_2_ on a bridge-site due to the steric hindrance of the top sites. Ag, Au, and Cu, on the other hand, show a comparable dissociation mechanism with pre-adsorbed NH_3_ on the pristine surface, explaining the comparable MEP.

### 3.2. Pd_3_M Alloys

#### 3.2.1. Adsorption-Induced Surface Segregation for Pd_3_*M*(1 1 1)

The results for the pure metal above suggest that Pd- or Ru-rich surfaces are expected to show significant surface coverage of H and NH_3_ at relevant operating conditions, while the presence of significant amounts of Ag, Au, or Cu on the surface could be detrimental for membrane performance. As previously discussed [20,26,27], surface termination of the Pd_3_*M* alloys can be influenced by adsorption-induced surface segregation over time during operation. Hence, we next determine surface segregation effects in the presence of H and NH_3_.

**Figure 7:**
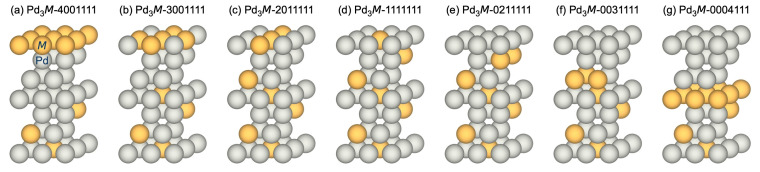
The different Pd_3_*M*(1 1 1) surface configuration investigated, (**a**) (4001111), (**b**) (3001111, (**c**) 2011111, (**d**) 1111111, (**e**) 0211111, (**f**) 0031111, and (**g**) 0004111. The numbers refer to the number of *M*-atoms per atomic layer (in total four atoms per layer), staring from the top surface layer where adsorption occurs. Grey atoms correspond to Pd; yellow atoms correspond to the alloying element.

In the following, we investigate seven different Pd_3_*M*(1 1 1) surface configurations illustrated in Figure 7, as described in Refs. [26,27]. The numbering refers to the number of alloy atoms (Ag, Au, Cu, Ru) in each layer of the slab (in total four atoms per layer), starting from the layer at which adsorption occurs.

**Figure 8:**
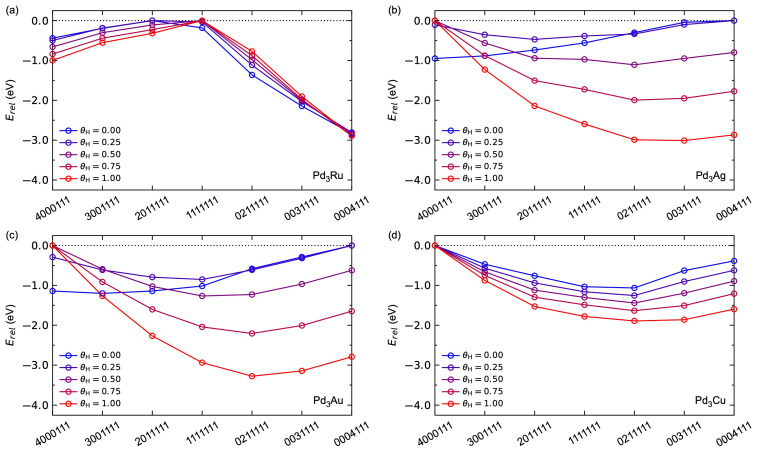
Calculated segregation energies as a function of hydrogen surface coverage *θ*_H_ for (**a**) Pd_3_Ru, (**b**) Pd_3_Ag, (**c**) Pd_3_Au, and (**d**) Pd_3_Cu.

We first focus on the surface segregation due to hydrogen adsorption. The relative energy for the seven configurations with respect to $\theta_{H}$ is shown in Figure 8. Pd_3_Ag and Pd_3_Au behave similarly. For low hydrogen surface coverages, Ag- and Au-rich surfaces are energetically most favoured. By increasing the hydrogen surface coverage, the Ag- and Au-terminated surfaces are destabilised relative to the Pd-terminated surfaces, where the (0211111) surface is the most stable at $\theta_{H}$ = 1, in line with previous work [26,27]. Pd_3_Cu shows a tendency towards Pd-terminated surfaces for all hydrogen coverages, where the (0211111) configuration is the most stable for all coverages investigated. Pd_3_Ru, on the other hand, shows a more complex segregation behaviour. For all coverages, the most stable [27,52,53] configuration is (0004111) with Ru deep into the subsurface of our slab model, where the relative stability is insensitive to $\theta_{H}.$ Interestingly, while the relative energies for (4000111), (3001111), and (2011111) become more negative with increasing $\theta_{H}$, the relative energies for (1111111), (0211111), and (0031111) become more positive with increasing $\theta_{H}$.

**Figure 9:**
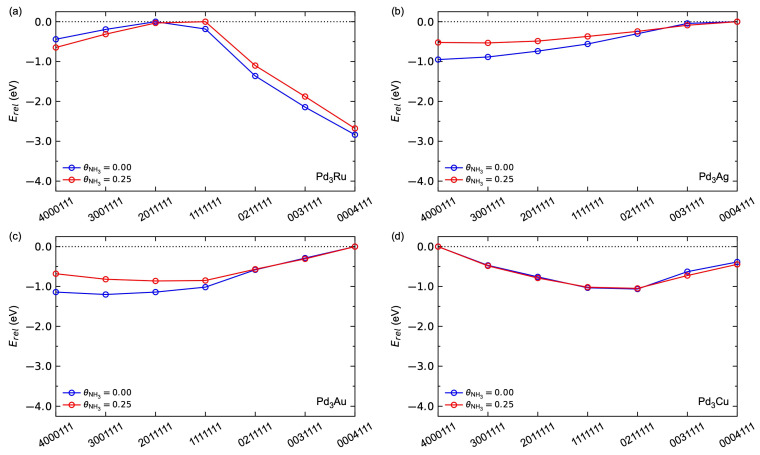
Calculated segregation energies as a function of NH_3_ surface coverage $\theta_{NH_{3}}$ for (**a**) Pd_3_Ru, (**b**) Pd_3_Ag, (**c**) Pd_3_Au, and (**d**) Pd_3_Cu.

Next, we focus on the segregation with respect to NH_3_ surface coverage shown in Figure 9. We observe a preference for Ag-rich surfaces at $\theta_{{NH}_{3}}$ = 0.25, in agreement with previous work [27]. As for the hydrogen adsorption described above, Pd_3_Au behaves in the same way as Pd_3_Ag. Note that we do observe a small relative destabilisation of the Ag- and Au-terminated surfaces with increasing $\theta_{{NH}_{3}}$, also apparent in our previous work on Pd_3_Ag [27]. Pd_3_Cu is found to be insensitive to $\theta_{{NH}_{3}}$. Pd_3_Ru again shows a more complex segregation effect, where the Ru-terminated surfaces show a relative increased stability with increasing $\theta_{{NH}_{3}}$, and the opposite is true for the Pd-terminated surface. Note, however, that the (0004111) configuration is the most stable for both $\theta_{{NH}_{3}}$ = 0 and $\theta_{{NH}_{3}}$ = 0.25 for Pd_3_Ru.

The results in Figure 8 and Figure 9 clearly indicate significant adsorption-induced surface segregation effects for the Pd_3_*M* alloys investigated. Since the surface Pd-to-*M* ratio is therefore expected to change gradually over time during operation, we will next assess the thermodynamics and kinetics as a function of surface *M*-content. In the following, we limit the study to the two extrema of fully Pd-covered or fully *M*-covered surfaces, as well as an intermediate model system with a nominal surface stoichiometry of Pd_3_*M*. For the Pd-terminated surfaces, we focus on the most stable surfaces under full hydrogen surface coverage, i.e., Pd_3_Ru (0004111), Pd_3_Ag (0211111), Pd_3_Au (0211111), and Pd_3_Cu (0211111). The *M*-terminated surfaces are modelled using the (4000111) configuration, while the Pd_3_*M*-terminated surfaces are modelled using the (1111111) configuration.

#### 3.2.2. Adsorption Thermodynamics

The calculated adsorption enthalpies $\Delta H^{ads}$, entropies $T\Delta S^{ads}$, energies $\Delta G^{ads}$, and vibrational frequencies for the different adsorbates on the Pd-terminated Pd_3_*M* surfaces are summarised in Table S1. All alloys show comparable adsorption thermodynamics, which is comparable to pure Pd. This is as expected, since the alloy surfaces are all Pd-terminated, and in line with previous work on Pd_3_Ag [27]. Note, however, that Pd_3_Cu show weaker H and NH_3_ adsorption. Ru and Cu show smaller DFT-calculated *fcc* lattice parameters of, respectively, 3.81 Å and 3.61 Å, compared to Pd (3.94 Å), while oppositely Ag and Au show larger *fcc* lattice parameters of, respectively, 4.14 Å and 4.16 Å, in line with the experimental observations [54]. Since alloying with larger *fcc* unit cell elements is found to lead to alloys with higher permeability [54], the changes in adsorption properties could also be due to the associated structural perturbations by substituting Pd with a different sized metal in the subsurface layer. Table 2 shows a summary of the calculated thermodynamics for the Pd_3_*M*-terminated Pd_3_*M* surfaces. We find a small decrease in the adsorption strength for H and NH_3_ for Pd_3_*M*-terminated Pd_3_Ag, Pd_3_Au, and Pd_3_Cu compared to the corresponding Pd-terminated surfaces in Table S1. For Pd_3_*M*-terminated Pd_3_Ru, we find a weaker adsorption of H and a stronger adsorption of NH_3_ compared to the corresponding Pd-terminated surface. Finally, the thermodynamics for the *M*-terminated Pd_3_*M* surfaces are summarised in Table S2. The thermodynamic properties are, as expected, comparable to the pure metals in Table 1.

**Figure 10:**
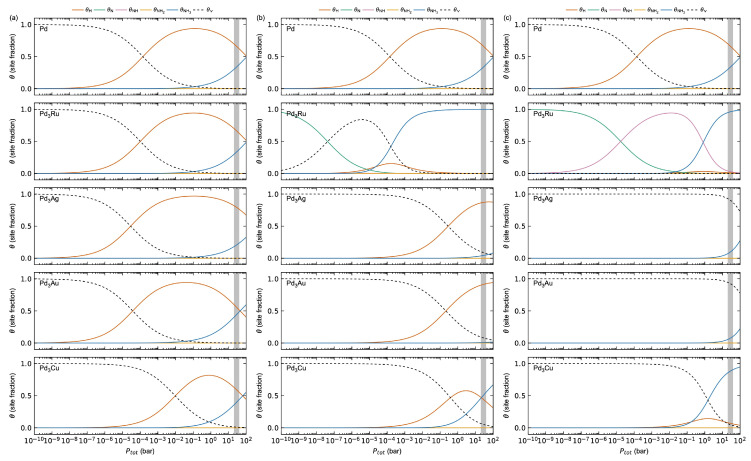
Calculated surface coverage on the (1 1 1) surfaces of Pd and (**a**) Pd-terminated, (**b**) Pd_3_*M*-terminated, and (**c**) *M*-terminated Pd_3_*M*, assuming a gas composition of $x_{NH_{3}}$ = $x_{N_{2}}$ = 0.2 and $x_{H_{2}}$ = 0.6 at *T* = 623 K. From top to bottom: Pd, Pd_3_Ru, Pd_3_Ag, Pd_3_Au, and Pd_3_Cu. Typical operating conditions for the Pd-alloy membranes of 20–40 bar are marked in grey.

The changes in thermodynamics with respect to the surface *M*-content indicate that an increase in surface *M*-content is expected to suppress the hydrogen surface coverage. The calculated surface coverages for H, N, NH, NH_2_, NH_3_, and empty surface sites (*v*) as a function of total pressure $P_{tot}$ at 623 K on the different Pd_3_*M* surfaces are plotted in Figure 10. As described above, we assume a gas mixture of 20% H_2_, 60% NH_3_, and 20% N_2_. The results for Pd in Figure 2a are also plotted in the top panels in Figure 10 for comparison.

Focusing first on Pd-terminated surfaces in Figure 10a, Pd_3_Ru, Pd_3_Ag, and Pd_3_Cu all behave comparably to Pd, as expected from the thermodynamics in Table 1 and Table S1; $\theta_{H}$ starts to increase for $P_{tot}$ > 10^−7^, with a maximum of $\theta_{H}$ ≈ 1 at $P_{tot}$ ≈ 10^−1^. For larger total pressures, $\theta_{NH_{3}}$ starts to increase at the expense of $\theta_{H}$. Pd_3_Cu shows a sharper shape for the hydrogen maximum, i.e., a narrower adsorption window, and a lower maximum value ($\theta_{H}$ ≈ 0.8), in line with the weaker hydrogen adsorption described above. The shape of the $\theta_{NH_{3}}$ curve, on the other hand, is comparable with Pd and the other alloys. By increasing the *M*-content (Figure 10b,c), the position of the maximum hydrogen surface coverage is shifted towards higher *P_tot_* for Pd_3_Ag, Pd_3_Au, and Pd_3_Cu. Interestingly, we find for Pd_3_Ru that the hydrogen coverage is significantly to fully suppressed for both the Pd_3_*M*- and *M*-terminated surfaces. This can be reasoned from the significant increase in the adsorption energy of NH_3_ in the presence of Ru on the surface. All Pd_3_Ag, Pd_3_Au, and Pd_3_Cu surfaces, as well as Pd-terminated Pd_3_Ru, show surface coverage only for H and NH_3_. Pd_3_*M*-terminated Pd_3_Ru show no surface coverage of NH, and none of the Pd_3_Ru surfaces show surface coverage of NH_2_.

**Figure 11:**
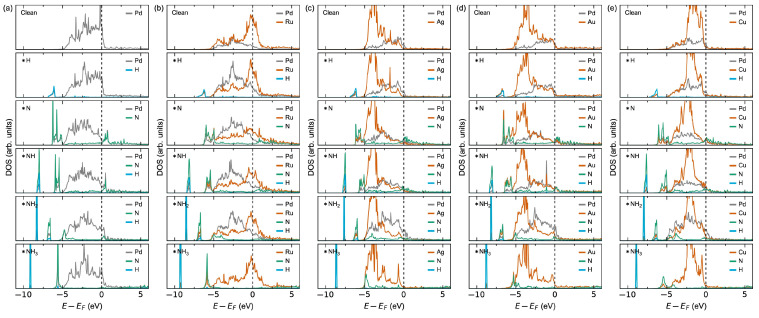
Atomically resolved electronic density of states (DOS) at the surface for a clean surface and with the adsorbates on (**a**) Pd and Pd_3_*M*-terminated (**b**) Pd_3_Ru, (**c**) Pd_3_Ag, (**d**) Pd_3_Au, and (**e**) Pd_3_Cu. Only the DOS for the adsorbates and the nearest metal surface atoms are shown. The DOS are scaled per atom.

The adsorption behaviour as a function of the surface *M*-content in Table 2, Tables S1 and S2 and Figure 10 can be further explained by the changes in electronic structure at the surface in Figure 11, Figures S1 and S2. As expected, all the Pd-terminated surfaces show comparable surface DOS to that of pure Pd (Figure S1), explaining their comparable adsorption behaviour. By increasing the surface *M*-content to Pd_3_*M*-termination (Figure 11) and *M*-termination (Figure S2), the surface DOS becomes increasingly more similar to their pure *M* counterparts, also as expected.

#### 3.2.3. Adsorbate–Adsorbate Interactions and Co-Adsorption

**Figure 12:**
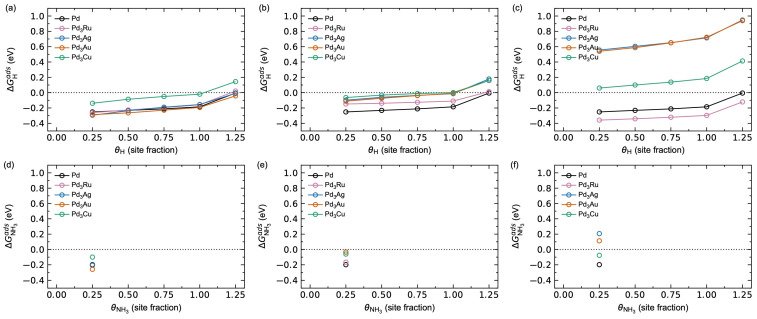
Calculated adsorption Gibbs energy for (**a**–**c**) H and (**d**–**f**) NH_3_ as a function of surface coverage *θ_i_* at *T* = 623 K and $p_{H_{2}}$ = $p_{{\mathrm{NH}}_{3}}$ = *p*^0^ = 1 bar on the surface of Pd and (**a**,**d**) Pd-terminated, (**b**,**e**) Pd_3_*M*-terminated, and (**c**,**f**) *M*-terminated Pd_3_*M*.

Figure 12 shows a comparison of the calculated H (a–c) and NH_3_ (d–f) adsorption energies as a function of increasing surface coverage $\theta_{i}$ for the different Pd_3_*M* surfaces investigated. The results for the Pd metal in Figure 5 are also plotted for comparison.

Pd-terminated Pd_3_Ru, Pd_3_Ag, and Pd_3_Au show comparable hydrogen adsorption energetics (Figure 12a,d), while Pd_3_Cu shows adsorption energies ~0.15 eV higher for all $\theta_{H}$, as expected from the adsorption thermodynamics in Table S1. In all cases, we find a hydrogen saturation coverage of $\theta_{H}$ = 1, with a sudden increase in $\Delta G_{H}^{ads}$ from $\theta_{H}$ = 1 to 1.25, attributed to the associated increased adsorbate–adsorbate interactions when the hcp sites also start to be occupied, as described above. The corresponding adsorption energies for NH_3_ on the Pd-terminated surfaces are plotted in Figure 12d. Increasing the surface *M*-content results in significant changes in the adsorption behaviour. Pd_3_Ru shows a small increase in $\Delta G_{H}^{ads}$ for Pd_3_*M*-termination (Figure 12b) and a decrease in $\Delta G_{H}^{ads}$ for *M*-termination (Figure 12c) relative to Pd-termination. Pd_3_Ag, Pd_3_Au, and Pd_3_Cu all show a destabilisation of hydrogen adsorption with increased surface *M*-content, most pronounced for Pd_3_Ag and Pd_3_Au. Similarly, Pd_3_Ru shows a stabilisation of NH_3_ adsorption with increasing surface *M*-content (Figure 12e,f), while Pd_3_Ag and Pd_3_Au show a destabilisation effect of NH_3_ adsorption. The adsorption of NH_3_ on Pd_3_Cu is insensitive to the surface *M*-content. Note that since the values for $\theta_{NH_{3}}$ = 0.25 for Pd-termination are comparable to those for Pd, we expect an NH_3_ saturation coverage of $\theta_{NH_{3}}$ = 0.25. Hence, we have not explicitly investigated larger NH_3_ coverages.

**Figure 13:**
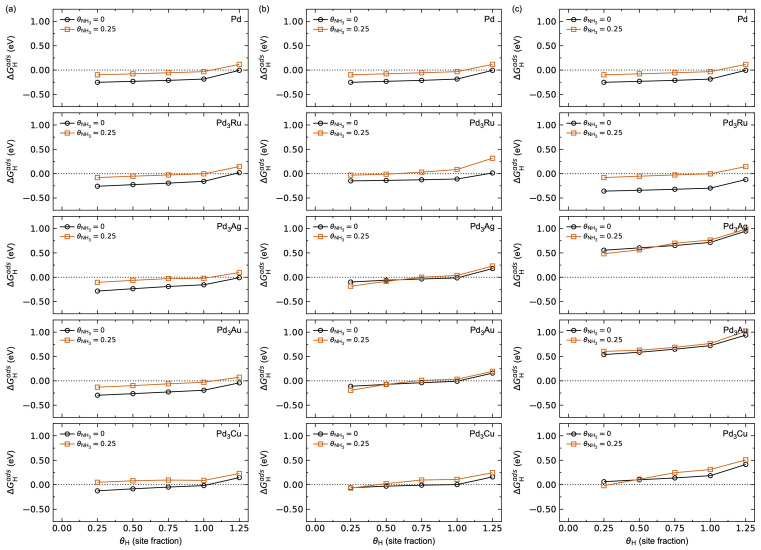
Calculated hydrogen adsorption energy for a clean surface (black) and with $\theta_{{\mathrm{NH}}_{3}}$ = 0.25 pre-adsorbed on the surface (red) of Pd and (**a**) Pd-terminated, (**b**) Pd_3_*M*-terminated, and (**c**) *M*-terminated Pd_3_*M*, as a function of hydrogen surface coverage *θ*_H_ at *T* = 623 K and $p_{H_{2}}$ = $p_{{\mathrm{NH}}_{3}}$ = *p*^0^ = 1 bar. From top to bottom: Pd, Pd_3_Ru, Pd_3_Ag, Pd_3_Au, and Pd_3_Cu.

The destabilisation effect for hydrogen adsorption with pre-adsorbed NH_3_ on the alloy surfaces is shown in Figure 13. For Pd-termination (Figure 13a), Pd_3_Ru, Pd_3_Ag, and Pd_3_Au behave comparable to Pd, while Pd_3_Cu shows a relative shift in the adsorption energies of ~0.15 eV. The corresponding expected hydrogen saturation coverage is $\theta_{H}$ = 1 for all Pd-terminated surfaces. By increasing the surface *M*-content (Figure 13b,c), two trends can be observed. Firstly, we observe the same trend in the (de)stabilisation of hydrogen adsorption with increasing surface *M*-content, as described above (Figure 12). Secondly, the destabilisation effect with respect to pre-adsorbed NH_3_ is less pronounced for Ag, Au, and Cu, attributed to their weaker NH_3_ adsorption (Table 2, Tables S1 and S2) and in line with the results for the pure metals (Table 1 and Figure 5).

#### 3.2.4. Hydrogen Dissociation Energetics

**Figure 14:**
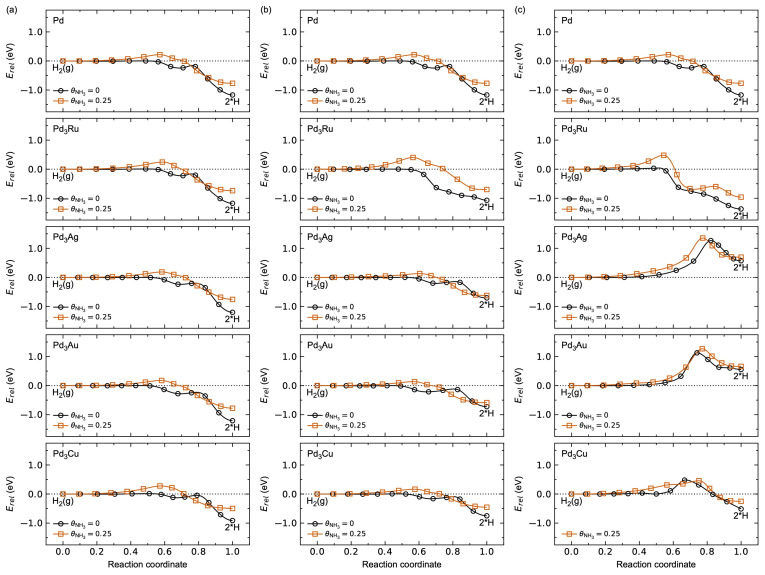
Calculated minimum energy path (MEP) for hydrogen dissociation for a clean surface (black) and with $\theta_{NH_{3}}$ = 0.25 pre-adsorbed on the surface (red) for Pd and (**a**) Pd-terminated, (**b**) Pd_3_*M*-terminated, and (**c**) *M*-terminated Pd_3_*M*. From top to bottom: Pd, Pd_3_Ru, Pd_3_Ag, Pd_3_Au, and Pd_3_Cu.

Finally, the calculated MEP for hydrogen dissociation without (black) and with NH_3_ pre-adsorbed on the surface (red) on Pd and the Pd_3_*M* alloy surfaces is plotted in Figure 14. For all alloy systems, we find that the H_2_ dissociation follows the same mechanism through a physiosorbed H_2_ on a top site, as for Pd. They also show comparable dissociation mechanisms through a physiosorbed H_2_ on a bridge-site due to the steric hindrance of the top sites, as described above.

Focusing first on the Pd-terminated surfaces (Figure 14a), we find a non-activated dissociation process on the clean alloy surfaces. Pd_3_Cu shows a less stable intermediate adsorbed H_2_ configuration, in line with the weaker adsorption energetics described above. NH_3_ present on the surface results in dissociation energy barriers of 0.24 eV, 019 eV, 0.18 eV, and 0.28 eV for Pd_3_Ru, Pd_3_Ag, Pd_3_Au, and Pd_3_Cu, respectively, comparable to that for Pd of 0.22 eV. For the fully *M*-covered surfaces (Figure 14c), the H_2_ dissociation without pre-adsorbed NH_3_ shifts towards an activated process for Pd_3_Ag, Pd_3_Au and Pd_3_Cu, while it remains a non-activated process for Pd_3_Ru, in line with the results for the pure metals in Figure 6. The H_2_ dissociation energy barriers become 1.27 eV, 1.13 eV, and 0.48 eV for *M*-terminated Pd_3_Ag, Pd_3_Au, and Pd_3_Cu, respectively. The corresponding H_2_ dissociation energy barriers with pre-adsorbed NH_3_ are 0.48 eV, 1.35 eV, 1.27 eV, and 0.46 eV for Pd_3_Ru, Pd_3_Ag, Pd_3_Au, and Pd_3_Cu, respectively. The dissociation energetics for Pd_3_*M*-termination (Figure 14b) are comparable to those for Pd-termination (Figure 14a), since the dissociation paths in all cases go through a surface Pd-site.

## 4. Conclusions

In this work, we have investigated hydrogen and ammonia co-adsorption phenomena on *M*(1 1 1) and Pd_3_*M*(1 1 1) (*M* = Pd, Ru, Ag, Au, Cu) surfaces using DFT calculations. We find a complex relation between adsorption properties with respect to surface metal stoichiometry and surface segregation, which is closely linked to the corresponding differences in electronic structure at the surface. For instance, while higher Ru-content on the surface is expected to give stronger hydrogen adsorption, hydrogen adsorption may still be hindered by competitive adsorption phenomena due to the stronger adsorption of NH_3_ and NH_3_-related species. The current results show that Pd metal or alloys with Pd-rich surfaces are expected to show the best surface hydrogen adsorption properties, including adsorption thermodynamics and hydrogen dissociation kinetics. Significant amounts of the alloying elements, Ru, Ag, Au, and Cu, investigated in this work tend to be detrimental for the surface hydrogen adsorption properties by either the destabilisation of hydrogen adsorption due to changes in electronic structure at the surface, the suppression of hydrogen adsorption due to the competitive adsorption of NH_3_, or the emergence of significant energy barriers for hydrogen dissociation at the surface due to either changes in electronic structure at the surface or steric hindrance in the presence of NH_3_.

## Figures and Tables

**Figure 1:**
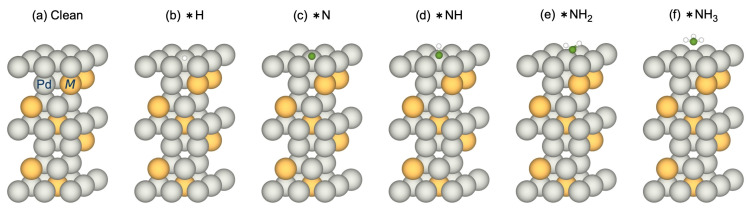
Representative calculated (1 1 1) surface atomic structures of Pd_3_Au for (**a**) a clean surface and the adsorption of (**b**) H, (**c**) N, (**d**) NH, (**e**) NH_2_, and (**f**) NH_3_. Gray and yellow atoms correspond to Pd and *M*, respectively.

**Table 1:** Calculated adsorption thermodynamics for H- and NH_3_-related species on the (1 1 1) metal surfaces at *T* = 623 K and $p_{H_{2}}$ = $p_{NH_{3}}$ = $p^{0}$ = 1 bar. The adsorption entropy of NH_3_ was obtained from the empirical relations in Ref. [35] (marked with *).

Species	Site	$\Delta H^{ads}$ (eV)	$T\Delta S^{ads}$ (eV)	$\Delta G^{ads}$ (eV)	$\nu_{i}$ (cm^−1^)
Pd					
H	fcc	−0.67	−0.42	−0.25	964, 841, 841
N	fcc	1.47	0.18	1.28	503, 494, 493
NH	fcc	0.60	−0.23	0.84	3377, 714, 714, 503, 437, 437
NH_2_	bridge	0.11	−0.59	0.70	3478, 3374, 1449, 673, 637, 575, 435, 320, 161
NH_3_	top	−0.84	−0.61 *	−0.20	-
Ru					
H	fcc	−0.66	−0.42	−0.24	1122, 823, 818
N	fcc	0.68	0.21	0.47	514, 384, 384
NH	fcc	−0.20	−0.22	0.01	3431, 693, 687, 572, 370, 368
NH_2_	bridge	−0.44	−0.59	0.15	3452, 3364, 1488, 674, 628, 620, 460, 307, 155
NH_3_	top	−0.92	−0.61	−0.31	-
Ag					
H	fcc	0.07	−0.40	0.47	799, 771, 771
N	fcc	3.97	0.22	3.75	396, 396, 395
NH	fcc	2.23	−0.18	2.41	3427, 555, 555, 398, 354, 354
NH_2_	bridge	0.81	−0.51	1.33	3511, 3409, 1481, 527, 502, 490, 350, 242, 112
NH_3_	top	−0.42	−0.61 *	0.19	-
Au					
H	fcc	0.03	−0.39	0.42	825, 678, 678
N	fcc	3.65	0.20	3.45	454, 454, 424
NH	fcc	2.20	−0.23	2.43	3445, 759, 759, 455, 455, 422
NH_2_	bridge	1.04	−0.57	1.61	3537, 3425, 1468, 693, 633, 582, 366, 282, 156
NH_3_	top	−0.41	−0.61 *	0.20	-
Cu					
H	fcc	−0.29	−0.42	0.14	1042, 854, 854
N	fcc	2.42	0.18	2.23	508, 482, 482
NH	fcc	0.99	−0.21	1.20	3449, 608, 607, 513, 408, 407
NH_2_	bridge	0.27	−0.55	0.82	3496, 3404, 1496, 575, 559, 552, 419, 287, 127
NH_3_	top	−0.49	−0.61 *	0.11	-

**Table 2:** Calculated adsorption thermodynamics for hydrogen and NH_3_-related species on the Pd_3_*M*-terminated (1 1 1) Pd_3_*M* surfaces at *T* = 623 K and $p_{H_{2}}$ = $p_{NH_{3}}$ = $p^{0}$ = 1 bar. The adsorption entropy of NH_3_ was obtained from the empirical relations in Ref. [35].

Species	Site	$\Delta H^{ads}$ (eV)	$T\Delta S^{ads}$ (eV)	$\Delta G^{ads}$ (eV)	$\nu_{i}$ (cm^−1^)
Pd_3_Ru					
H	fcc	−0.61	−0.41	−0.19	1076, 1074, 431
N	fcc	0.62	0.21	0.41	566, 531, 134
NH	fcc	0.01	−0.21	0.23	3375, 725, 538, 488, 442, 281
NH_2_	bridge	−0.42	−0.59	0.17	3493, 3385, 1447, 647, 564, 430, 426, 209, 119
NH_3_	top	−1.16	−0.61	−0.56	-
Pd_3_Ag					
H	fcc	−0.45	−0.40	−0.05	1076, 1074, 431
N	fcc	2.53	0.24	2.29	566, 531, 134
NH	fcc	1.43	−0.20	1.63	3375, 725, 538, 488, 442, 281
NH_2_	bridge	0.63	−0.53	1.16	3493, 3385, 1447, 647, 564, 430, 426, 209, 119
NH_3_	top	−0.48	−0.61	0.13	-
Pd_3_Au					
H	fcc	−0.47	−0.41	−0.06	964, 920, 662
N	fcc	2.36	0.19	2.17	511, 502, 378
NH	fcc	1.36	−0.22	1.58	3390, 715, 684, 479, 430, 396
NH_2_	bridge	0.67	−0.57	1.23	3508, 3398, 1446, 627, 607, 568, 411, 271, 146
NH_3_	top	−0.39	−0.61	0.22	-
Pd_3_Cu					
H	fcc	−0.46	−0.42	−0.04	1045, 898, 740
N	fcc	2.23	0.19	2.04	517, 492, 419
NH	fcc	1.05	−0.22	1.27	3393, 701, 665, 490, 425, 403
NH_2_	bridge	0.36	−0.57	0.93	3478, 3379, 1462, 603, 590, 526, 415, 300, 149
NH_3_	top	−0.64	−0.61	−0.03	-

## Data Availability

The data presented in this study are available on request from the corresponding author.

## Supplementary Materials

The following supporting information can be downloaded at: https://www.mdpi.com/article/10.3390/membranes15050135/s1, Table S1: Calculated adsorption thermodynamics on Pd-terminated Pd_3_*M*; Table S2: Calculated adsorption thermodynamics on *M*-terminated Pd_3_*M*; Table S3: Calculated adsorption thermodynamics on Pd using different functionals; Figure S1: Electronic density of states for adsorption on Pd-terminated Pd_3_*M*; Figure S2. Electronic density of states for adsorption on *M*-terminated Pd_3_*M*.

## References

[1] Kurien C., Mittal M. (2023). Utilization of green ammonia as a hydrogen energy carrier for decarbonization in spark ignition engines. Int. J. Hydrogen Energy.

[2] Langella G., De Joannon M., Sabia P., Iodice P., Amoresano A. (2022). Ammonia as a fuel for internal combustion engines: Latest advances and future challenges. J. Phys. Conf. Ser..

[3] Qi Y., Liu W., Liu S., Wang W., Peng Y., Wang Z. (2023). A review on ammonia-hydrogen fueled internal combustion engines. eTransportation.

[4] Tornatore C., Marchitto L., Sabia P., De Joannon M. (2022). Ammonia as Green Fuel in Internal Combustion Engines: State-of-the-Art and Future Perspectives. Front. Mech. Eng..

[5] Valera-Medina A., Amer-Hatem F., Azad A.K., Dedoussi I.C., De Joannon M., Fernandes R.X., Glarborg P., Hashemi H., He X., Mashruk S. (2021). Review on Ammonia as a Potential Fuel: From Synthesis to Economics. Energy Fuels.

[6] Rathore S.S., Biswas S., Fini D., Kulkarni A.P., Giddey S. (2021). Direct ammonia solid-oxide fuel cells: A review of progress and prospects. Int. J. Hydrogen Energy.

[7] Dhawale D.S., Biswas S., Kaur G., Giddey S. (2023). Challenges and advancement in direct ammonia solid oxide fuel cells: A review. Inorg. Chem. Front..

[8] Cheliotis M., Boulougouris E., Trivyza N.L., Theotokatos G., Livanos G., Mantalos G., Stubos A., Stamatakis E., Venetsanos A. (2021). Review on the Safe Use of Ammonia Fuel Cells in the Maritime Industry. Energies.

[9] Dimitriou P., Javaid R. (2020). A review of ammonia as a compression ignition engine fuel. Int. J. Hydrogen Energy.

[10] Keller M., Koshi M., Otomo J., Iwasaki H., Mitsumori T., Yamada K. (2020). Thermodynamic evaluation of an ammonia-fueled combined-cycle gas turbine process operated under fuel-rich conditions. Energy.

[11] Jeerh G., Zhang M., Tao S. (2021). Recent progress in ammonia fuel cells and their potential applications. J. Mater. Chem. A.

[12] Miyaoka H., Miyaoka H., Ichikawa T., Ichikawa T., Kojima Y. (2018). Highly purified hydrogen production from ammonia for PEM fuel cell. Int. J. Hydrogen Energy.

[13] Okanishi T., Okura K., Srifa A., Muroyama H., Matsui T., Kishimoto M., Saito M., Iwai H., Yoshida H., Saito M. (2017). Comparative Study of Ammonia-fueled Solid Oxide Fuel Cell Systems. Fuel Cells.

[14] García-García F.R., Ma Y.H., Rodríguez-Ramos I., Guerrero-Ruiz A. (2008). High purity hydrogen production by low temperature catalytic ammonia decomposition in a multifunctional membrane reactor. Catal. Commun..

[15] Collins J.P., Way J.D. (1994). Catalytic decomposition of ammonia in a membrane reactor. J. Membr. Sci..

[16] Itoh N., Oshima A., Suga E., Sato T. (2014). Kinetic enhancement of ammonia decomposition as a chemical hydrogen carrier in palladium membrane reactor. Catal. Today.

[17] Cechetto V., Di Felice L., Medrano J.A., Makhloufi C., Zuniga J., Gallucci F. (2021). H2 production via ammonia decomposition in a catalytic membrane reactor. Fuel Process. Technol..

[18] Cechetto V., Agnolin S., Di Felice L., Pacheco Tanaka A., Llosa Tanco M., Gallucci F. (2023). Metallic Supported Pd-Ag Membranes for Simultaneous Ammonia Decomposition and H2 Separation in a Membrane Reactor: Experimental Proof of Concept. Catalysts.

[19] Zhang J., Xu H., Li W. (2006). High-purity COx-free H2 generation from NH3 via the ultra permeable and highly selective Pd membranes. J. Membr. Sci..

[20] Peters T.A., Polfus J.M., Stange M., Veenstra P., Nijmeijer A., Bredesen R. (2016). H 2 flux inhibition and stability of Pd-Ag membranes under exposure to trace amounts of NH 3. Fuel Process. Technol..

[21] Zhang Z., Liguori S., Fuerst T.F., Way J.D., Wolden C.A. (2019). Efficient Ammonia Decomposition in a Catalytic Membrane Reactor To Enable Hydrogen Storage and Utilization. ACS Sustain. Chem. Eng..

[22] Cechetto V., Di Felice L., Gallucci F. (2023). Advances and Perspectives of H_2_ Production from NH_3_ Decomposition in Membrane Reactors. Energy Fuels.

[23] Clark D., Malerød-Fjeld H., Budd M., Yuste-Tirados I., Beeaff D., Aamodt S., Nguyen K., Ansaloni L., Peters T., Vestre P.K. (2022). Single-step hydrogen production from NH_3_, CH_4_, and biogas in stacked proton ceramic reactors. Science.

[24] Peters T.A., Polfus J.M., van Berkel F.P.F., Bredesen R. (2016). Interplay between propylene and H2S co-adsorption on the H2 flux characteristics of Pd-alloy membranes employed in propane dehydrogenation (PDH) processes. Chem. Eng. J..

[25] Svenum I.-H., Herron J.A., Mavrikakis M., Venvik H.J. (2020). Pd3Ag(111) as a Model System for Hydrogen Separation Membranes: Combined Effects of CO Adsorption and Surface Termination on the Activation of Molecular Hydrogen. Top. Catal..

[26] Svenum I.-H., Herron J.A., Mavrikakis M., Venvik H.J. (2012). Adsorbate-induced segregation in a PdAg membrane model system: Pd3Ag(111). Catal. Today.

[27] Småbråten D.R., Strømsheim M.D., Peters T. (2024). Hydrogen and NH3 co-adsorption on Pd–Ag membranes. Int. J. Hydrogen Energy.

[28] Blöchl P.E. (1994). Projector augmented-wave method. Phys. Rev. B.

[29] Kresse G., Furthmüller J. (1996). Efficient iterative schemes for ab initio total-energy calculations using a plane-wave basis set. Phys. Rev. B.

[30] Kresse G., Joubert D. (1999). From ultrasoft pseudopotentials to the projector augmented-wave method. Phys. Rev. B.

[31] Perdew J.P., Burke K., Ernzerhof M. (1996). Generalized Gradient Approximation Made Simple. Phys. Rev. Lett..

[32] Henkelman G., Uberuaga B.P., Jónsson H. (2000). A climbing image nudged elastic band method for finding saddle points and minimum energy paths. J. Chem. Phys..

[33] Henkelman G., Jónsson H. (2000). Improved tangent estimate in the nudged elastic band method for finding minimum energy paths and saddle points. J. Chem. Phys..

[34] Chase M.W. (1998). NIST-JANAF Thermochemical Tables, Fourth Edition. J. Phys. Chem. Ref. Data Monogr..

[35] Campbell C.T., Sellers J.R.V. (2012). The Entropies of Adsorbed Molecules. J. Am. Chem. Soc..

[36] Carabineiro S.A.C., Matveev A.V., Gorodetskii V.V., Nieuwenhuys B.E. (2004). Selective oxidation of ammonia over Ru(0001). Surf. Sci..

[37] Danielson L.R., Dresser M.J., Donaldson E.E., Dickinson J.T. (1978). Adsorption and desorption of ammonia, hydrogen, and nitrogen on ruthenium (0001). Surf. Sci..

[38] Liu R., Shen W., Zhang J., Li M. (2008). Adsorption and dissociation of ammonia on Au(111) surface: A density functional theory study. Appl. Surf. Sci..

[39] Jiang Z., Qin P., Fang T. (2014). Mechanism of ammonia decomposition on clean and oxygen-covered Cu (1 1 1) surface: A DFT study. Chem. Phys..

[40] Kulkarni S.R., Realpe N., Yerrayya A., Velisoju V.K., Sayas S., Morlanes N., Cerillo J., Katikaneni S.P., Paglieri S.N., Solami B. (2023). Elucidating the rate-determining step of ammonia decomposition on Ru-based catalysts using ab initio-grounded microkinetic modeling. Catal. Sci. Technol..

[41] Perdew J.P., Ruzsinszky A., Csonka G.I., Vydrov O.A., Scuseria G.E., Constantin L.A., Zhou X., Burke K. (2008). Restoring the Density-Gradient Expansion for Exchange in Solids and Surfaces. Phys. Rev. Lett..

[42] Hammer B., Hansen L.B., Nørskov J.K. (1999). Improved adsorption energetics within density-functional theory using revised Perdew-Burke-Ernzerhof functionals. Phys. Rev. B.

[43] Zhang Y., Yang W. (1998). Comment on “Generalized Gradient Approximation Made Simple”. Phys. Rev. Lett..

[44] Furness J.W., Kaplan A.D., Ning J., Perdew J.P., Sun J. (2020). Accurate and Numerically Efficient r^2^ SCAN Meta-Generalized Gradient Approximation. J. Phys. Chem. Lett..

[45] Grimme S., Ehrlich S., Goerigk L. (2011). Effect of the damping function in dispersion corrected density functional theory. J. Comput. Chem..

[46] Hamada I. (2014). Van der Waals density functional made accurate. Phys. Rev. B Condens. Matter Mater. Phys..

[47] Klimeš J., Bowler D.R., Michaelides A. (2010). Chemical accuracy for the van der Waals density functional. J. Phys. Condens. Matter.

[48] Klimeš J., Bowler D.R., Michaelides A. (2011). Van der Waals density functionals applied to solids. Phys. Rev. B.

[49] Polfus J.M., Peters T., Bredesen R., Løvvik O.M. (2021). Vacancy diffusion in palladium hydrides. Phys. Chem. Chem. Phys..

[50] Hammer B., Nørskov J.K. (2000). Theoretical surface science and catalysis—Calculations and concepts. Advances in Catalysis.

[51] Karimadom B.R., Sermiagin A., Meyerstein D., Zidki T., Mizrahi A., Bar-Ziv R., Kornweitz H. (2024). Hydrogen adsorption on various transition metal (111) surfaces in water: A DFT forecast. Phys. Chem. Chem. Phys..

[52] Dong W., Hafner J. (1997). H2 dissociative adsorption on Pd(111). Phys. Rev. B.

[53] Dipojono H.K., Padama A.A.B., Ozawa N., Nakanishi H., Kasai H. (2010). A First Principles Study on Dissociation and Adsorption Processes of H2 on Pd3Ag(111) Surface. Jpn. J. Appl. Phys..

[54] Peters T.A., Kaleta T., Stange M., Bredesen R. (2011). Development of thin binary and ternary Pd-based alloy membranes for use in hydrogen production. J. Membr. Sci..

