# Supplementary material for: Hydrogen and Ammonia Co-Adsorption on M(1 1 1) and Pd3M(1 1 1) (M = Pd, Ru, Ag, Au, Cu) Surfaces
Source: Membranes (Basel). 2025 May 1;15(5):135. doi: 10.3390/membranes15050135 (PMC12113646; doi:10.3390/membranes15050135)
Supplement: Supplementary file 1 [file membranes-15-00135-s001.zip › membranes-3568591-supplementary.pdf]

Supplementary information

Hydrogen and Ammonia Co-adsorption on *M*(1 1 1) and Pd_3*M*(1 1 1) (*M* = Pd, Ru, Ag, Au, Cu) Surfaces

Didrik R. Småbråten[1,*], Marie D. Strømsheim[2] and Thijs A. Peters[1]

[1]Department of Sustainable Energy Technology, SINTEF Industry, 0314 Oslo, Norway

[2]Hydrogen Mem-Tech AS, 7038 Trondheim, Norway

* E-mail: didrik.smaabraaten@sintef.no

Table S1. Calculated adsorption thermodynamics for hydrogen and NH_3 related species on the Pd-terminated (1 1 1) Pd_3M surfaces at T = 623 K and $p_{H_2} = p_{NH_3} = p^0$ = 1 bar. The adsorption entropy of NH_3 was obtained from the empirical relations in Ref. [35] in the main text (marked with *).

Species	Site	ΔH^{ads} (eV)	$T\Delta S^{ads}$ (eV)	ΔG^{ads} (eV)	ν_i (cm^{-1})
Pd_3Ru					
H	fcc	-0.68	-0.42	-0.26	1006, 845, 826
N	fcc	1.55	0.18	1.36	515, 486, 483
NH	fcc	0.67	-0.23	0.90	3379, 717, 716, 512, 430, 429
NH_2	bridge	0.14	-0.59	0.72	3479, 3374, 1447, 667, 629, 575, 436, 309, 162
NH_3	top	-0.81	-0.61*	-0.21	-
Pd_3Ag					
H	fcc	-0.71	-0.42	-0.29	930, 879, 837
N	fcc	1.63	0.19	1.45	504, 488, 464
NH	fcc	0.70	-0.23	0.93	3367, 728, 711, 481, 430, 426
NH_2	bridge	0.13	-0.58	0.71	3475, 3374, 1453, 672, 629, 560, 427, 316, 154
NH_3	top	-0.81	-0.61*	-0.21	-
Pd_3Au					
H	fcc	-0.70	-0.42	-0.29	923, 868, 818
N	fcc	1.54	0.18	1.36	501, 495, 474
NH	fcc	0.66	-0.23	0.89	3366, 721, 706, 482, 425, 423
NH_2	bridge	0.09	-0.58	0.68	3472, 3373, 1451, 681, 634, 554, 428, 315, 155
NH_3	top	-0.81	-0.61*	-0.21	-
Pd_3Cu					
H	fcc	-0.56	-0.42	-0.14	1042, 862, 775
N	fcc	1.95	0.19	1.76	515, 477, 470
NH	fcc	1.02	-0.22	-0.10	3383, 687, 672, 512, 408, 401

NH_2	bridge	0.33	-0.58	0.91	3489, 3385, 1456, 637, 609, 571, 426, 285, 157
NH_3	top	-0.71	-0.61*	-0.10	-

Table S2. Calculated adsorption thermodynamics for hydrogen and NH_3 related species on the *M*-terminated (1 1 1) Pd_3M surfaces at T = 623 K and $p_{H_2} = p_{NH_3} = p^0$ = 1 bar. The adsorption entropy of NH_3 was obtained from the empirical relations in Ref. [35] in the main text (marked with *).

Species	Site	ΔH^{ads} (eV)	$T\Delta S^{ads}$ (eV)	ΔG^{ads} (eV)	ν_i (cm^{-1})
Pd_3Ru					
H	fcc	-0.77	-0.41	-0.36	1001, 784, 780
N	fcc	-0.04	0.20	-0.24	484, 412, 412
NH	fcc	-0.77	-0.22	-0.55	3405, 742, 737, 539, 375, 373
NH_2	bridge	-0.86	-0.62	-0.24	3453, 3363, 1476, 756, 705, 626, 477, 346, 178
NH_3	top	-1.19	-0.61*	-0.59	-
Pd_3Ag					
H	fcc	0.15	-0.40	0.55	928, 704, 671
N	fcc	4.32	0.23	4.09	439, 343, 343
NH	fcc	2.45	-0.16	2.62	3436, 509, 508, 439, 319, 319
NH_2	bridge	0.88	-0.51	1.39	3516, 3413, 1480, 509, 498, 480, 361, 225, 109
NH_3	top	-0.40	-0.61*	0.21	-
Pd_3Au					
H	fcc	0.14	-0.40	0.54	967, 648, 639
N	fcc	3.97	0.20	3.77	468, 417, 417
NH	fcc	2.66	-0.20	2.86	3443, 616, 613, 457, 387, 384
NH_2	bridge	1.19	-0.55	1.74	3545, 3426, 1456, 635, 580, 544, 368, 230, 155
NH_3	top	-0.38	-0.61*	0.23	-
Pd_3Cu					
H	fcc	-0.36	-0.42	0.06	980, 848, 817
N	fcc	2.23	0.19	2.03	476, 475, 451
NH	fcc	0.64	-0.21	0.86	3414, 665, 664, 473, 407, 405

NH_2	bridge	-0.04	-0.57	0.53	3465, 3383, 1499, 638, 593, 533, 418, 315, 133
NH_3	top	-0.67	-0.61*	-0.06	-

Table S3. Comparison of calculated adsorption thermodynamics using different functionals for H and NH_3 on the Pd (1 1 1) surfaces at T = 623 K and $p_{\mathrm{H_2}}$ = $p_{\mathrm{NH_3}}$ = p^0 = 1 bar. The adsorption entropy of NH_3 was obtained from the empirical relations in Ref. [35] in the main text (marked with *).

Species	Site	ΔH^{ads} (eV)	$T\Delta S^{ads}$ (eV)	ΔG^{ads} (eV)	ν_i (cm^{-1})
PBE					
H	fcc	-0.67	-0.42	-0.25	964, 841, 841
NH_3	top	-0.81	-0.61*	-0.20	-
PBEsol					
H	fcc	-0.85	-0.42	-0.43	1027, 870, 867
NH_3	top	-1.07	-0.61*	-0.46	-
RPBE					
H	fcc	-0.52	-0.42	-0.10	933, 822, 820
NH_3	top	-0.54	-0.61*	0.07	-
revPBE					
H	fcc	-0.54	-0.42	-0.12	942, 827, 826
NH_3	top	-0.55	-0.61*	0.06	-
R2SCAN					
H	fcc	-0.74	-0.42	-0.32	977, 845, 845
NH_3	top	-0.96	-0.61*	-0.36	-
DFT-D3(BJ)					
H	fcc	-0.73	-0.42	-0.31	1010, 844, 844
NH_3	top	-1.10	-0.61*	-0.49	-
rev-vdW-DF2					
H	fcc	-0.65	-0.42	-0.23	981, 822, 822
NH_3	top	-1.06	-0.61*	-0.45	-

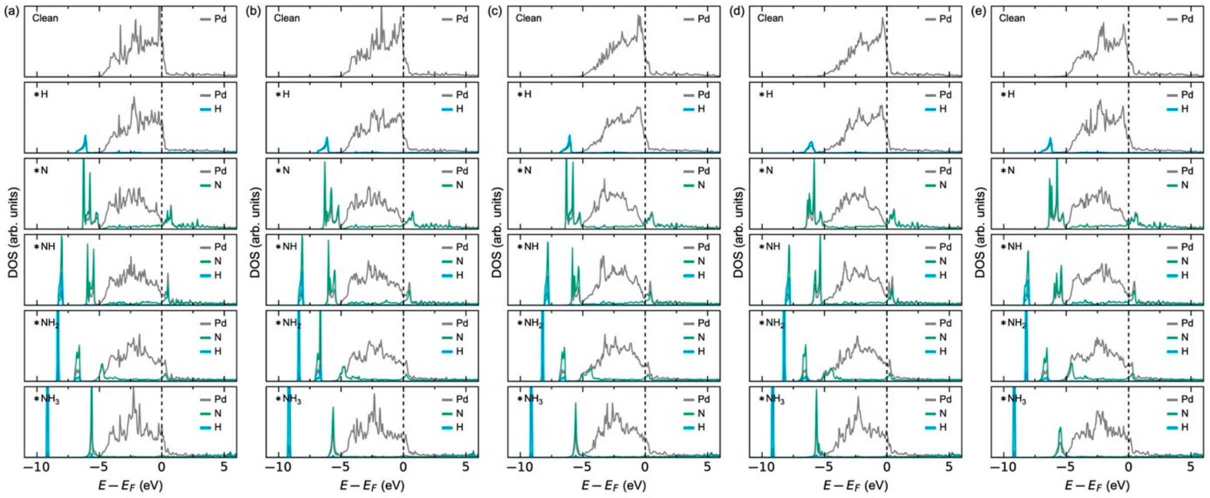

Figure S1. Atomically resolved electronic density of states (DOS) at the surface for a clean surface and with the adsorbates on (a) Pd, and Pd-terminated (b) Pd_3Ru, (c) Pd_3Ag, (d) Pd_3Au and (e) Pd_3Cu. Only the DOS for the adsorbates and the nearest metal surface atoms are shown. The DOS are scaled per atom.

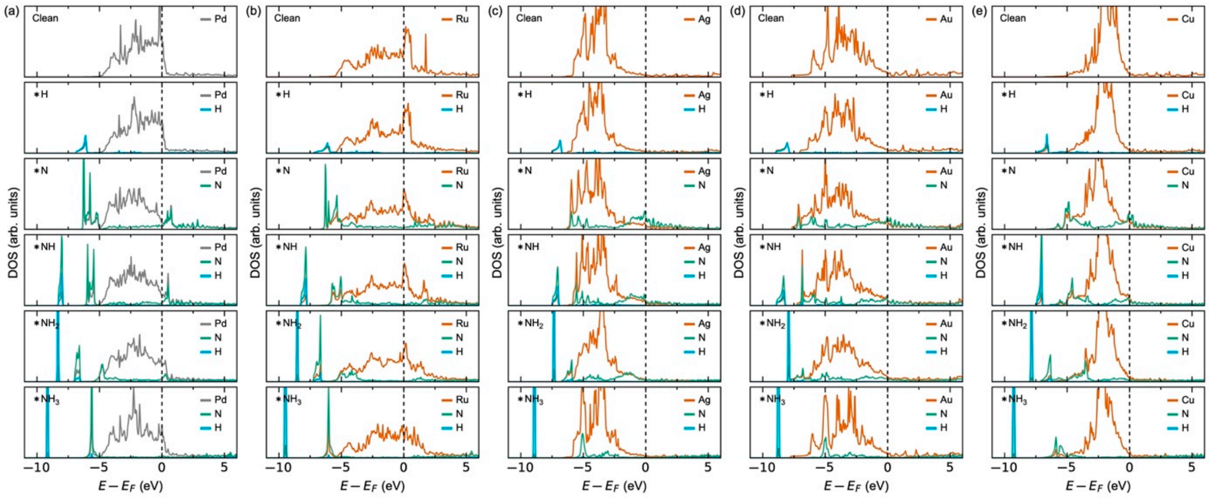

Figure S2. Atomically resolved electronic density of states (DOS) at the surface for a clean surface and with the adsorbates on (a) Pd, and *M*-terminated (b) Pd_3Ru, (c) Pd_3Ag, (d) Pd_3Au and (e) Pd_3Cu. Only the DOS for the adsorbates and the nearest metal surface atoms are shown. The DOS are scaled per atom.
